# Quality of life across chemotherapy lines in patients with cancers of the pancreas and biliary tract

**DOI:** 10.1186/1471-2407-12-390

**Published:** 2012-09-06

**Authors:** August Zabernigg, Johannes M Giesinger, Georg Pall, Eva-Maria Gamper, Klaus Gattringer, Lisa M Wintner, Monika J Sztankay, Bernhard Holzner

**Affiliations:** 1 Department of Internal Medicine, Kufstein County Hospital, Endach 27, A-6330, Kufstein, Austria; 2 Department of Psychiatry and Psychotherapy, Innsbruck Medical University, Anichstr.35, A-6020, Innsbruck, Austria; 3 Department of Internal Medicine, Innsbruck Medical University, Anichstr.35, A-6020, Innsbruck, Austria; 4 Leopold-Franzens-University of Innsbruck, Innrain 52, A-6020, Innsbruck, Austria

**Keywords:** Quality of life, Pancreatic cancer, Cancer of biliary tract, Chemotherapy, Electronic patient-reported outcome monitoring

## Abstract

**Background:**

In patients with cancers of the pancreatic and biliary tract quality of life (QOL) improvement is the main treatment goal, since survival can be prolonged only marginally. Up to date, knowledge on QOL impairments throughout the entire treatment process, often including several chemotherapy lines, is scarce. Our study aimed at investigating QOL trajectories from adjuvant treatment to palliative 3^rd^-line therapy

**Methods:**

Patients were included in routine electronic patient-reported outcome monitoring at Kufstein County Hospital at the time of diagnosis and assessed with the EORTC QLQ-C30 during each chemotherapy cycle.

**Results:**

Eighty out of 147 patients with pancreatic cancer or cancer of the bile ducts treated at the Kufstein County Hospital, fulfilled inclusion criteria and could be included in the study (mean age 67.4 years; 53.8% women). Physical, Emotional and Cognitive Functioning, and Global QOL deteriorated across chemotherapy lines, whereas Fatigue, Pain, Dyspnoea, Sleeping Disturbances, Diarrhoea, and Taste Alterations increased. With regard to Physical Functioning, Global QOL, Fatigue, Dyspnoea, Diarrhoea and Taste Alterations, the patients receiving adjuvant or 1^st^-line palliative chemotherapy did not differ significantly. Most patients in 2^nd^- or 3^rd^-line chemotherapy showed significantly higher impairments and symptom burden. However, patients under 1^st^ and 2^nd^-line treatment showed stable QOL trajectories, whereas 3^rd^-line patients perceived substantial deteriorations.

**Conclusions:**

The results suggest early palliative treatment initiation to stabilise QOL on a level as high as possible. The continuous QOL improvement during adjuvant treatment, probably reflecting post-operative recovery, may indicate that deleterious effects of adjuvant chemotherapy on QOL are highly unlikely.

## Background

Despite advances in palliative treatment, locally advanced and metastatic carcinoma of the pancreas and bile ducts are still associated with an extremely poor prognosis.

As chemotherapy results in only a marginal prolongation of survival, quality of life (QOL) improvement is usually referred to as the main treatment goal [1-3].

For patients with metastatic pancreatic cancer, different 1^st^-line treatments can be chosen according to performance status, age, co-morbidities and the patient´s preference (Gemcitabine, Gemcitabine/Erlotinib, FOLFIRINOX) [2,4,5]. However, none of the combination therapies thus far have been superior to Gemcitabine monotherapy in improving QOL [6-10]. For patients with cancers of the biliary tract and gall bladder who receive palliative chemotherapy, a combination of Gemcitabine and Cisplatin has been widely accepted as a standard 1^st^-line treatment [11].

In the 2^nd^-line setting, the combination of 5-FU/Leucovorin/Oxaliplatin (OFF) has shown a survival benefit in a phase III randomised trial [12]. Therefore, in clinical practice, many patients currently receive more than one line of treatment. This is also true for patients with bile duct tumours, although the evidence for 2^nd^-line treatment from clinical trials is less robust [13].

Almost all studies on QOL and the clinical benefit rate, however, refer to 1^st^-line therapy. Knowledge of the longitudinal course of QOL across the different treatment lines is almost completely lacking, even though such information might be important for medical decision making and patient information [14-16]. Furthermore, although adjuvant chemotherapy with Gemcitabine has now become an accepted treatment standard after surgical resection for early pancreatic cancer, there is minimal information on the impact of this treatment on QOL [17].

As with other tumours, patient-reported outcomes (PRO) provide useful prognostic information in patients with pancreatic cancer and cancer of the biliary tract [3,18]. Thus, the objective of our study was to analyse and compare patient-reported QOL and physical/psychosocial symptom burden, measured by repeated computer-assisted completion of validated questionnaires in patients with pancreatic cancer and cancer of the bile ducts, from adjuvant treatment to palliative 3^rd^-line therapy.

## Methods

### Sample

Patients were included in routine electronic PRO monitoring at the Department of Internal Medicine of the Kufstein County Hospital at the time of diagnosis and assessed, when possible, during each chemotherapy cycle. The inclusion criteria were as follows: diagnosis of pancreatic cancer or cancer of the bile ducts, no severe cognitive impairments, and the ability to speak German.

The data collection was performed by a study nurse who approached the patient to obtain informed consent and administer the PRO questionnaire. The patients entered the data themselves on a tablet-PC that presented the QLQ-C30 questionnaire while the study nurse was available for any help required. We used the Computer-based Health Evaluation System (CHES)[19] to record electronic PRO data.

Clinical and sociodemographic data were collected from the hospital records and entered in the CHES database to match PRO data. The study was approved by the ethics committee of Innsbruck Medical University.

### PRO Assessment - EORTC QLQ-C30

The EORTC QLQ-C30 [20] is an internationally validated and widely used questionnaire to assess QOL, psychosocial burden and physical symptoms in cancer patients. It consists of five functioning scales (Physical, Social, Role, Cognitive, and Emotional functioning), a scale for Global QOL, and nine symptom scales (Fatigue, Pain, Nausea/Vomiting, Dyspnoea, Appetite Loss, Sleep Disturbance, Constipation, Diarrhoea and Financial Difficulties).

Two additional items asking for taste alterations were taken from the EORTC Quality of Life Group item bank (“Have you had problems with your sense of taste?” and “Did food and drink taste different from usual?”). These items were summed to the taste alteration score, which has already been used in previous studies [21,22]. The score ranges from 0 to 100 points, with higher values indicating more severe taste alterations.

### Statistical Analysis

Patient characteristics are presented as means, standard deviations, and percentages. Group comparisons of patient baseline characteristics were performed with Chi^2^ tests and T-tests for independent samples, to investigate whether chemotherapy line comparisons should be adjusted for age, sex, marital status, and type of cancer (gall bladder and pancreatic). We did not consider adjusting for variables such as time since diagnosis, previous surgery, occurrence of metastasis, or chemotherapy regimen, as these variables reflect genuine characteristics of the chemotherapy line.

To compare the symptom burden and functioning between patients in different chemotherapy lines, we used mixed linear models, including the following terms: a random baseline, a first-order autocorrelation covariance matrix, and a main effect patient group (chemotherapy line). A major advantage of mixed linear models is that they allow for data modelling with a varying number of assessments per patient. Additionally, these models allow time-varying co-variates, such as chemotherapy line.

In a secondary analysis, we investigated the symptom burden and functioning trajectories within chemotherapy lines using the time since the start of the chemotherapy line as the main effect in separate mixed linear models. Additionally, we compared patients during the 1^st^ and 2^nd^-line palliative chemotherapy with regard to changes in symptom burden and functioning, between the start of the chemotherapy line and the time points of tumour staging (6–8 weeks and 12–16 weeks after the start of chemotherapy, respectively). For this analysis, we dichotomised patients into non-responders (progressive disease) and responders (stable disease or remission).

As a measure of changes in symptom burden or functioning, we provide monthly change rates for the EORTC QLQ-C30 scales, i.e., change in score points per 30 days. For ease of interpretation, these numbers should be related to the thresholds for minimal important change. According to Osoba et al. [23], for the QLQ-C30, a change of 5–10 score points indicates a small clinical change, while 10–20 indicates a moderate change, and above 20 points is a large change.

All statistical analyses were performed with SPSS 20.0.

## Results

### Patient characteristics

Between May 2005 and September 2011, 147 patients with pancreatic cancer or cancer of the bile ducts were treated at the Kufstein County Hospital (110 patients with pancreatic cancer and 37 patients with cancer of the bile ducts, which includes patients with cancer of the gall bladder). Eighty of these patients could be included in ePROM, with a total number of PRO-assessments of 771 (on average 9 assessments per patient, SD 7.4), i.e., an inclusion rate of 54.4%.

The reasons for not including a patient were as follows: treatment with surgery only, rejection of chemotherapy by the patient, reduced performance status and, in a few cases, rejection of PRO assessment by the patient or basic language problems.

The mean age at baseline was 67.4 years (SD 9.6), with 53.8% of the patients being female. Of the total number of patients, 72.5% (n = 58) were suffering from pancreatic cancer and 27.5% (n = 22) from cancer of the bile ducts, including a few patients with cancer of the gall bladder. At the time of study inclusion, 29.1% of the patients received adjuvant chemotherapy, 68.4% received 1^st^-line palliative chemotherapy, and 2.5% received 2^nd^-line chemotherapy (for these few patients, no 1^st^-line PRO data were available).

In Table 1, patient characteristics are given separately for patients receiving different chemotherapy lines. During the study period, 58.2% of the patients received one chemotherapy line, 25.3% received two chemotherapy lines, and 16.5% received three or four chemotherapy lines.

**Table 1:** Sociodemographic and clinical patient characteristics*

		**Adjuvant CT n=23**	**1**^**st**^**line pall n=65**	**2**^**nd**^**line pall n=29**	**3**^**rd**^**+ line pall n=8**	**p**
**Age**	mean (SD)	66.6 (8.2)	66.9 (10.0)	64.5 (10.8)	60.9 (14.6)	0.370
Sex	men	34.8%	52.3%	65.5%	50.0%	0.182
	women	65.2%	47.7%	34.5%	50.0%	
Diagnosis	pancreatic cancer	78.3%	72.3%	75.9%	87.5%	0.786
	gall cancer	21.7%	27.7%	24.1%	12.5%	
Time since diagnosis^§^	mean (SD)	3.4 (5.0)	3.5 (4.5)	11.1 (7.5)	24.8 (8.1)	<0.001
Metastasis	yes	17.4%	48.3%	62.1%	87.5%	0.001
	no	82.6%	51.7%	37.9%	12.5%	
Previous Surgery	yes	100.0%	58.9%	70.4%	71.4%	0.004
	no	0.0%	41.1%	29.6%	28.6%	
Chemotherapy regimen	Gemcitabine Mono	90.9%	40.6%	6.9%	0.0%	<0.001
	Gemcitabine Combination Therapy	9.1%	45.3%	27.6%	25.0%	
	FOLFIRINOX	0.0%	4.7%	0.0%	0.0%	
	FOLFOX/XELOX	0.0%	9.4%	55.2%	37.5%	
	FOLFIRI	0.0%	0.0%	3.4%	12.5%	
	Other	0.0%	0.0%	6.9%	25.0%	

Total N = 80, whereas 36 patients were accounted for two, six patients for three and three patients for three or more chemotherapy lines because they passed from one line to another.

^§^ Number of months that passed since diagnosis, averaged across all assessments within a chemotherapy line.

In the adjuvant setting, almost all patients with pancreatic cancer received Gemcitabine monotherapy. Only in rare cases of intolerance to Gemcitabine was 5-FU-based chemotherapy applied.

Palliative 1^st^-line therapy consisted of Gemcitabine monotherapy (40.6%) and Gemcitabine-based doublets (45.3%; Capecitabine, Oxaliplatin and, occasionally, Cisplatin). FOLFIRINOX was only recently introduced as a palliative 1^st^-line treatment option; therefore, only a few patients were treated with this regimen.

As 2^nd^-line therapies, patients usually received FOLFOX (55.2%) or a Gemcitabine-based doublet (27.6%). Only a few patients were treated with FOLFIRI, Docetaxel or Gemcitabine combinations in the 3^rd^-line setting.

**Figure 1:**
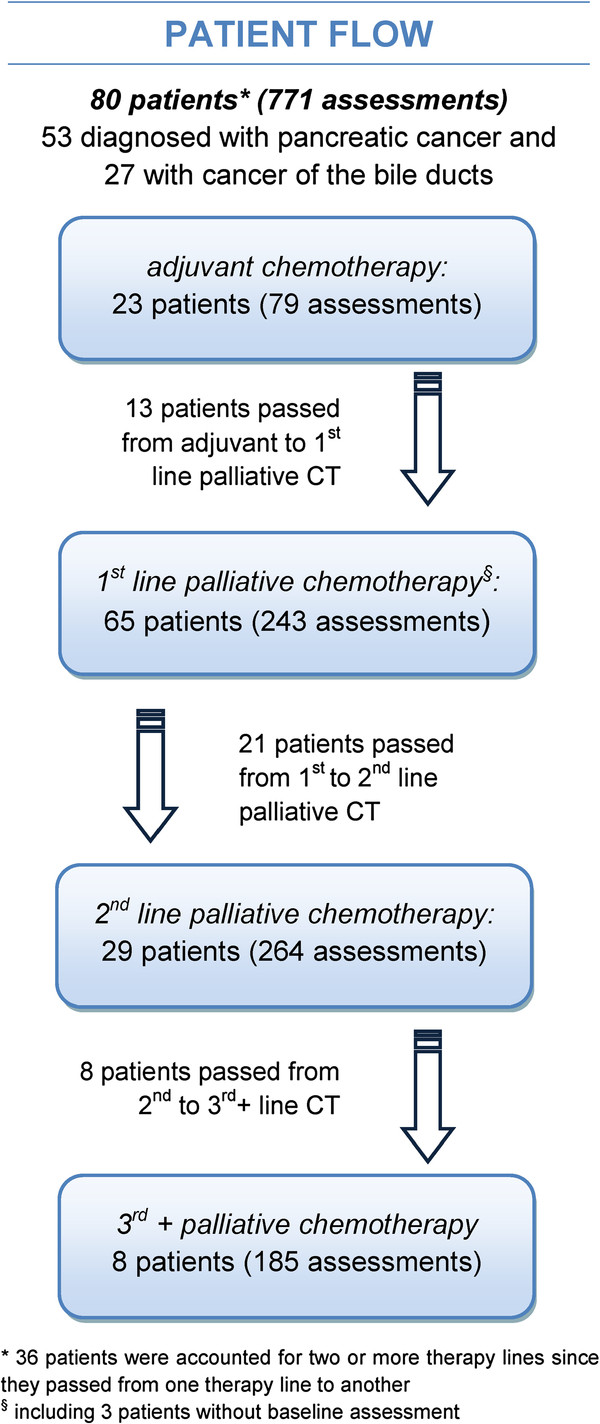
Patient flow concerning chemotherapy lines.

Within the study period, 16.5% of the patients passed from adjuvant to palliative chemotherapy, 26.5% passed from 1^st^- to 2^nd^-line palliative chemotherapy and 10% passed from 2^nd^- to 3^rd^+-line palliative chemotherapy, for further details see Figure 1.

### Differences in symptom burden between chemotherapy lines

To investigate the differences between the chemotherapy lines with regard to patient-reported physical and psychosocial symptom burden, we compared patients during adjuvant chemotherapy, 1^st-^line palliative chemotherapy, 2^nd^-line palliative chemotherapy, and 3^rd^-line palliative chemotherapy or beyond. As expected, we found a significant association between chemotherapy line and Physical, Emotional and Cognitive Functioning, Global QOL, Fatigue, Pain, Dyspnoea, Sleeping Disturbances, Diarrhoea, and Taste Alterations with worse outcomes in later treatment lines. Role and Social Functioning, Nausea/Vomiting, Appetite Loss, Constipation and Financial Impact could not be related to chemotherapy lines.

For pairwise comparisons, we provide point differences for the smallest and the largest significant difference. Using pairwise comparisons, we found that, with regard to Physical Functioning, Global QOL, Fatigue, Dyspnoea, Diarrhoea and Taste Alterations, the patients receiving adjuvant or 1^st^-line palliative chemotherapy did not differ significantly. However, most patients in 2^nd^- or 3^rd^-line chemotherapy showed significantly higher impairments and symptom burden.

**Figure 2:**
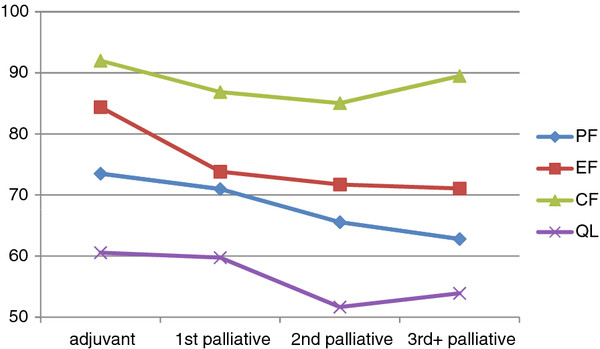
Comparison of functioning and Global QOL between chemotherapy lines (PF=Physical Functioning, EF=Emotional Functioning, CF=Cognitive Functioning, QL=Global Quality of Life).

**Figure 3:**
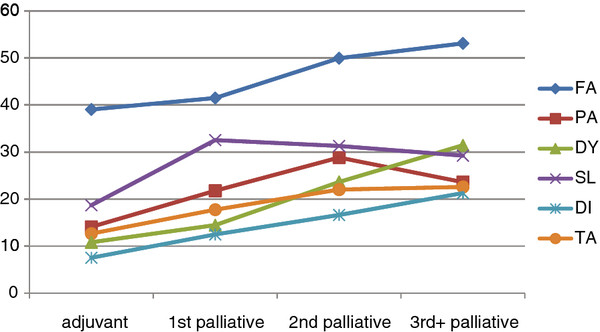
Comparison of symptom burden between chemotherapy lines (FA=Fatigue, PA=Pain, DY=Dyspnoea, SL=Sleeping Disturbance, DI=Diarrhoea, TA=Taste Alteration).

With regard to Emotional and Cognitive Functioning, Pain, and Sleeping Disturbances, the patients who received adjuvant chemotherapy were less affected than patients receiving any line of palliative chemotherapy. Due to the limited number of patients with third- or later-line chemotherapy, pairwise comparisons for this patient group had only limited statistical power. Therefore, certain absolute differences emerged as statistically non-significant, even though they were rather large. For further details see Table 2, Figure 1: Patient flow concerning chemotherapy lines, Figure 2, and Figure 3.

**Table 2:** Comparisons of physical and psychosocial symptom burden between chemotherapy lines.

**EORTC QLQ-C30**	**(0)**	**(1)**	**(2)**	**(3)**	**p**
	**Adjuvant CT**	**1st pall. CT**	**2nd pall. CT**	**3rd+pall. CT**	
Physical Functioning	73.5^2,3^	71.0^2^	65.6^0,1^	62.8^0^	0.036
Role Functioning	58.0	58.4	53.1	53.1	0.438
Social Functioning	79.0	74.4	74.8	69.6	0.145
Emotional Functioning	84.4^1,2,3^	73.8^0^	71.7^0^	71.1^0^	<0.001
Cognitive Functioning	92.0^1,2^	86.9^0^	85.1^0^	89.5	0.034
Global QOL	60.6^2^	59.7^2^	51.7^01^	53.9	0.002
Fatigue	39.1^2,3^	41.5^2,3^	50.0^0,1^	53.1^0,1^	0.004
Pain	14.1^1,2,3^	21.8^0,2^	28.8^0,1^	23.6^0^	<0.001
Nausea/Vomiting	7.6	13.2	12.2	11.0	0.128
Dyspnoea	10.8^2,3^	14.5^2,3^	23.6^0,1,3^	31.4^0,1,2^	<0.001
Appetite Loss	25.6	28.6	32.8	38.8	0.194
Sleeping Disturbances	18.7^1,2,3^	32.5^0^	31.3^0^	29.2^0^	0.001
Constipation	19.4	17.9	12.0	14.3	0.173
Diarrhoea	7.5^2,3^	12.5^3^	16.6^0^	21.3^0,1^	0.002
Financial Impact	10.8	11.3	12.0	12.8	0.946
Taste Alterations	12.7^2,3^	17.8	22.0^0^	22.6^0^	0.046

Superscript numbers indicate significant differences to other groups in pairwise comparisons.

### Symptom trajectories within chemotherapy lines

In a secondary analysis, we investigated the physical and psychosocial symptom trajectories (i.e., change rates) within chemotherapy lines. During adjuvant chemotherapy, a significant improvement was found for Physical Functioning (+3.3 score points per month), Role Functioning (+6.8), Emotional Functioning (+1.8), Global QOL (+4.1), Fatigue (−3.4) and Pain (−2.4).

During 1^st^- and 2^nd^-line palliative chemotherapy, symptom burdens remained stable, with the exception of Taste Alterations, which increased by +1.8 points per month during 1^st^-line chemotherapy, and Cognitive Functioning, which deteriorated by −1.2 points per month during 2^nd^-line chemotherapy.

During 3^rd^-line or higher palliative chemotherapy, the following symptoms and functioning aspects deteriorated significantly: Physical Functioning (−3.0), Role Functioning (−3.2), Social Functioning (−2.1), Emotional Functioning (−2.1), Global QOL (−1.6), Fatigue (+3.3), Dyspnoea (+4.6), Sleeping Disturbances (+3.0), and Financial Impact of Disease (+2.4). Further details are given in Table 3.

**Table 3:** Change in physical and psychosocial symptom burden within chemotherapy lines

**EORTC QLQ-C30**	**Adjuvant CT**		**1st pall. CT**		**2nd pall. CT**		**3**^**rd**^**+pall. CT**	
	**monthly change rate**	**p**	**monthly change rate**	**p**	**monthly change rate**	**p**	**monthly change rate**	**p**
**Physical** Functioning	3.3	<0.001	−0.1	0.868	−1.2	0.087	−3.0	0.001
Role Functioning	6.8	<0.001	0.0	0.994	−1.7	0.059	−3.2	0.002
Social Functioning	1.9	0.135	1.1	0.071	−1.1	0.110	−2.1	0.019
Emotional Functioning	1.8	0.006	0.6	0.301	−0.5	0.299	−2.3	0.001
Cognitive Functioning	−0.1	0.901	0.1	0.859	−1.2	0.036	−1.4	0.200
Global QOL	4.1	<0.001	0.0	0.955	−0.9	0.166	−1.6	0.012
Fatigue	−3.4	0.009	0.0	0.992	1.3	0.117	3.3	0.002
Pain	−1.1	0.128	−0.1	0.902	0.0	0.952	0.6	0.394
Nausea/Vomiting	−2.4	0.006	−0.9	0.162	−0.6	0.450	2.0	0.105
Dyspnoea	−0.5	0.494	0.4	0.476	0.6	0.506	4.6	0.006
Appetite Loss	−1.8	0.067	−0.2	0.802	−0.7	0.341	3.0	0.030
Sleeping Disturbances	−4.3	0.022	−1.2	0.182	0.9	0.358	2.0	0.055
Constipation	−1.7	0.217	−1.2	0.171	−0.5	0.380	−1.0	0.202
Diarrhoea	0.0	0.986	−0.4	0.445	0.9	0.221	0.1	0.911
Financial Impact	0.7	0.593	0.5	0.322	0.4	0.550	2.4	0.017
Taste Alterations	1.9	0.123	1.8	0.012	1.1	0.136	−0.3	0.749

* Monthly change rates give change estimates in score points per 30 days, based on the mixed effect model.

An additional analysis of patients during 1^st^ and 2^nd^-line palliative chemotherapy showed statistically significant differences with regard to symptom and functioning trajectories (i.e., differences in monthly change rates), according to the chemotherapy response with regard to the following scales: Physical Functioning (responders +0.9 points per month vs. non-responders −5.3 points per month), Role Functioning (+1.9 vs. -10.4), Global QOL (+1.6 vs. -4.7), Fatigue (−1.8 vs. +5.7 points).

## Discussion

Since the publication of the study by Glimelius et al., palliative chemotherapy, when compared with the best supportive care alone, is usually considered to improve the QOL and survival of patients with metastatic pancreatic cancer and cancer of the bile ducts, respectively [1]. In a consecutive trial reported by Burris et al., Gemcitabine monotherapy appeared to be superior to 5-FU in terms of the clinical benefit rate and survival and therefore established a new standard of care for advanced pancreatic cancer [2]. A large number of trials evaluating Gemcitabine-based combinations produced negative results or only marginal improvement concerning survival, and the data on the improvement of QOL were controversial [7-9,24].

Thus far, QOL assessment has focused on palliative 1^st^-line treatment, but there is still a paucity of data on the degree of improvement in QOL that can be achieved by chemotherapy [14,16]. In addition, there is a large uncertainty on the differences concerning the QOL aspects of patients in different treatment lines and on the QOL effects of adjuvant chemotherapy after surgery with curative intent.

Frequent, computer-assisted assessments of the EORTC-QLQ-C30 during adjuvant and palliative treatment in daily clinical practice, as presented in our analysis, may shed light on some of these questions.

Most importantly, and in accordance with other studies, our data indicate that patients under 1^st^-line palliative chemotherapy experience a stabilisation of physical and psychosocial symptoms and global QOL, rather than a real measurable improvement. These data, which suggest QOL stabilisation, are in line with the results reported by Romanus et al., who treated patients with Gemcitabine or Gemcitabine/Bevacizumab [6]. As in our analysis, these treatments resulted in a modest improvement in pain and mood and a slight slowing of functional deterioration but no significant improvement of global QOL. Furthermore, the most effective chemotherapy protocol, FOLFIRINOX, has also been shown to only slow the deterioration of QOL instead of significantly improving it, despite prolonging survival [4].

In an exploratory analysis with limited statistical power, we attempted to correlate the chemotherapy response, defined as disease stabilisation on CT-scans at 6–8 weeks after the start of chemotherapy, with QOL data. Our data indicate the importance of achieving disease-stabilisation, at the least, as these patients also seem to accomplish significant gains in important aspects of QOL.

Only recently, based on a small phase III trial, palliative chemotherapy with 5-FU/Folinic Acid/Oxaliplatin (OFF) has become the standard of care in the 2^nd^-line setting [12]. The QOL data were not reported in this trial. A number of phase II studies have also reported a measurable effect of 2^nd^-line chemotherapy [16,25]. However, due to infrequent assessments and the associated low compliance, QOL data have rarely been reported. As in the 1^st^-line palliative setting, the QOL trajectories shown by 2^nd^-line palliative chemotherapy patients indicate a stabilisation, rather than an improvement, of physical and psychosocial symptoms and global QOL.

These results are of clinical importance because they indicate a comparable effect of palliative chemotherapy on many of the aspects of QOL under 1^st^- and 2^nd^-line palliative chemotherapy in terms of constant QOL data during treatment. When stabilisation, but not a significant improvement of QOL, is obtainable with palliative chemotherapy, treatment should be initiated as soon as possible, when the clinical symptom burden is still low.

The comparison of QOL outcomes during different chemotherapy lines, as presented here, suggests that most of the QOL aspects show a progressive deterioration during the course of the disease over the chemotherapy lines. Though 2^nd^- and 3^rd^-line palliative chemotherapy patients reported higher symptom burden than adjuvant and 1^st^-line palliative chemotherapy patients, only 1^st^- and 2^nd^-line palliative chemotherapy patients showed stable QOL trajectories in contrast to 3^rd+^-line patients, who reported considerable QOL deteriorations over time.

In that regard, the outcomes for patients receiving adjuvant chemotherapy are of great interest. In contrast to the palliative situation, our data indicate that during the course of adjuvant chemotherapy, most of the subscales of QOL show a continuous improvement, probably caused by the fading of postoperative health problems. According to our analysis, deleterious effects of adjuvant chemotherapy on QOL seem highly unlikely. This information is of immediate clinical relevance in cases where adjuvant chemotherapy is suggested to our patients.

Comparing different studies on QOL in patients with cancer of the pancreas or bile ducts has some limitations, especially when different instruments and questionnaires are used. In our opinion, one of the major problems of assessing QOL in these rapidly progressive cancers is the timing and frequency of the assessments. We have no knowledge about the optimal frequency, but when too much time elapses between two surveys, we cannot detect rapid but clinically important changes in QOL [8,16,24].

The computer-assisted assessment of QOL data in daily clinical practice, as used in our analysis, offers important advantages over QOL assessments that have long time intervals, which are presented in most clinical studies. The computer-assisted assessments enabled us to evaluate QOL at many time points with short intervals, allowing a more robust estimate of longitudinal QOL changes during each treatment course. For patients with aggressive tumours, such as pancreatic cancer, usually with limited efficacy of palliative systemic treatments, QOL assessments are of even greater importance.

Certainly QOL has always to be seen as a multifactorial process and patients’ subjectively perceived QOL is influenced by a variety of factors. Patients experience positive and negative effects also due to their status, social and family support and individual coping strategies. Chemotherapy especially ameliorates the tumor associated symptoms but it is only one way to improve quality of life of patients with advanced cancer. A recent study, conducted by Temel et al., in patients with newly diagnosed metastatic non-small-cell lung cancer demonstrated impressively that early palliative care intervention in concert with standard oncology care improves QOL and median survival and reduces depressive symptoms, compared with patients receiving only standard treatment [26]. Besides treatment possibilities, high socioeconomic status also has been found to influence overall cancer survival positively [27,28] and social support was linked to cancer survival as well, although the results are divergent [29]. Lately, Cavalli-Björkman et al. reported that colorectal cancer patients, who did not live in a joint household with their significant other, were irrespective of age and co-morbidity less likely to receive combination chemotherapy and had poorer survival [30]. Social support in terms of rehabilitation programs in a setting of 12 weeks resulted in better QOL and improved physical functioning [31], whereas a 6-day residential psychosocial course did not show any effects [32]. Taken these findings together, it is obvious that a lot of research still has be done to identify influential factors on patients’ QOL, and how they relate to each other.

Our analysis has some limitations. As the number of patients included is relatively low, the statistical power is limited. Furthermore, some heterogeneity exists in the evaluated population, which concerns important clinical factors and the applied systemic treatments. Nevertheless, the patients included in this study represent our daily clinical practice.

## Conclusion

In conclusion, our data indicate that the frequent, computer-assisted assessment of QOL in patients with pancreatic cancer or cancer of the biliary tract results in important information that suggests QOL stabilisation as the main effect of chemotherapy in the palliative setting during different treatment lines and excluding the relevant deterioration of QOL during adjuvant chemotherapy. Therefore, we recommend these procedures to be included in future clinical trials to provide more robust information on QOL effects of different therapeutic interventions.

## Competing interest

The authors declare that they have no competing interests.

## Authors’ contributions

AZ, JMG, GP and BH conceived the study and participated in its design. AZ and KG coordinated data collection. JMG and EMG were involved in statistical analysis and AZ, GP, JMG; BH, EMG, KG, LMW and MJS in data interpretation. AZ, JMG, GP, BH, LMW, and MJS helped to draft the manuscript. All authors read andapproved the final manuscript.

## Pre-publication history

The pre-publication history for this paper can be accessed here:

http://www.biomedcentral.com/1471-2407/12/390/prepub

## Author details

^1^Department of Internal Medicine, Kufstein County Hospital, Endach 27, A-6330, Kufstein, Austria. ^2^Department of Psychiatry and Psychotherapy, Innsbruck Medical University, Anichstr.35, A-6020, Innsbruck, Austria.^3^Department of Internal Medicine, Innsbruck Medical University, Anichstr.35, A-6020, Innsbruck, Austria. ^4^Leopold-Franzens-University of Innsbruck, Innrain 52, A-6020, Innsbruck, Austria.
